# Pellet diameter of *Ganoderma lucidum* in a repeated-batch fermentation for the trio total production of biomass-exopolysaccharide-endopolysaccharide and its anti-oral cancer beta-glucan response

**DOI:** 10.3934/microbiol.2020023

**Published:** 2020-10-22

**Authors:** Nur Raihan Abdullah, Faez Sharif, Nur Hafizah Azizan, Ismail Fitri Mohd Hafidz, Sugenendran Supramani, Siti Rokhiyah Ahmad Usuldin, Rahayu Ahmad, Wan Abd Al Qadr Imad Wan-Mohtar

**Affiliations:** 1 Functional Omics and Bioprocess Development Laboratory, Institute of Biological Sciences, Faculty of Science, Universiti of Malaya, 50603, Kuala Lumpur, Malaysia; 2 Department of Biotechnology, Kulliyyah of Science, International Islamic University Malaysia, 25200 Kuantan, Pahang, Malaysia; 3 Agro-Biotechnology Institute, Malaysia (ABI), National Institutes Biotechnology Malaysia (NIBM), C/O HQ MARDI, 43400, Serdang, Selangor, Malaysia; 4 Halal Action Laboratory, Kolej Genius Insan, University Sains Islam Malaysia, Bandar Baru Nilai, 71800, Nilai, Negeri Sembilan, Malaysia; 5 Bioscience Research Institute, Athlone Institute of Technology, Ireland

**Keywords:** *Ganoderma lucidum*, pellet morphology, repeated-batch fermentation, exopolysaccharide, endopolysaccharide, anti-oral cancer

## Abstract

The pellet morphology and diameter range (DR) of *Ganoderma lucidum* were observed in a repeated-batch fermentation (RBF) for the trio total production of biomass, exopolysaccharide (EPS) and endopolysaccharide (ENS). Two factors were involved in RBF; broth replacement ratio (BRR: 60%, 75% and 90%) and broth replacement time point (BRTP: log, transition and stationary phase) in days. In RBF, 34.31 g/L of biomass favoured small-compact pellets with DR of 20.67 µm< d < 24.00 µm (75% BRR, day 11 of BRTP). EPS production of 4.34 g/L was prone to ovoid-starburst pellets with DR of 34.33 µm< d <35.67 µm (75% BRR, day 13 of BRTP). Meanwhile, the highest 2.43 g/L of ENS production favoured large-hollow pellets with DR of 34.00 µm< d < 38.67 µm (90% BRR, day 13 of BRTP). In addition, RBF successfully shortened the biomass-EPS–ENS fermentation period (31, 33 and 35 days) from batch to 5 days, in seven consecutive cycles of RBF. In a FTIR detection, β-glucan (BG) from EPS and ENS extracts were associated with β-glycosidic linkages (2925 cm^−1^, 1635 cm^−1^, 1077 cm^−1^, 920 cm^−1^ and 800 cm^−1^ wavelengths) with similar ^1^H NMR spectral behaviour (4.58, 3.87 and 3.81 ppm). Meanwhile, 4 mg/L of BG gave negative cytotoxic effects on normal gingival cell line (hGF) but induced antiproliferation (IC_50_ = 0.23 mg/mL) against cancerous oral Asian cellosaurus cell line (ORL-48). Together, this study proved that *G. lucidum* mycelial pellets could withstand seven cycles of long fermentation condition and possessed anti-oral cancer beta-glucan, which suits large-scale natural drug fermentation.

## 1. Introduction

Fermentation strategies have been unchanged for decades [1]. The conventional way to study mushrooms was through solid-state fermentation (SSF) until submerged liquid fermentation (SLF) was improved [2]. SLF caught researchers' attention due to its high yield of biomass and polysaccharide production in a shorter time period and with lower costs compared to SSF [3]. There are many ways to perform SLF, but the most promising production of a bioactivity compound from mushroom has been seen in repeated-batch fermentation (RBF) [4]. RBF is an adjustment of batch fermentation, in which a specific amount of medium is extracted and replaced by the same amount of medium extracted with a new medium, either intermittently, or more than once without changing the culture.

Predominantly, RBF is performed using bacteria, however, a study conducted by Wan-Mohtar et al., 2016 [5] has shown that RBF can also be used for basidiomycetes fungus, especially for *Ganoderma lucidum* (GL). GL is one of the broadly utilized species in biochemical and pharmaceutical fields [6], to produce ganoderic acid and polysaccharide for medicinal purposes. GL was also considered as a ‘remedy that could resuscitate the dead’ [7], and has been utilized for the prevention and treatment of numerous sorts of maladies; it is accepted to have anti-cancer and anti-ageing properties and is hostile to microbial or viral capacities [8],[9].

Extensive studies have been conducted on GL for its production of polysaccharides, due to its high medicinal properties. A further study conducted by Wan-Mohtar et al., 2016 [10] proved that the production of polysaccharide is directly affected by the morphology of mycelial pellets. Small-loosely branched mycelium pellets produce higher polysaccharide compared to large mycelium pellets [4]. In this study, the ideal pellet diameters of GL strain QRS 5120 were reported for the highest total biomass, exopolysaccharide and endopolysaccharide productions, as well as its anti-oral β-glucan (BG) bioactive properties.

Reports have been made regarding the cytotoxic effects of GL in vivo and in vitro where the GL extracts have demonstrated the effect in various lines of cancer cells, including the breast, pancreas, lung, colon and prostate [11]. To the best of our knowledge, only limited studies have demonstrated preliminary evidence of GL extract having a cytotoxic effect and inhibition activity toward oral cancer cells [12]. Hence, in this work, β-glucan (BG) from GL was screened on normal oral cell lines (gingival cell line; hGF) as well as a malignant oral cancer (cancerous oral Asian cellosaurus cell line; ORL-48) from an infected Malaysian patient, via 3-(4,5-dimethylthiazol-2-yl)-2,5-diphenyl tetrazolium bromide (MTT) colourimetric assay. MTT cytotoxicity assay is widely used to test inhibitor sensitivity to cultured cells, as it is an economical and rapid test that does not require animal model use.

## 2. Material and methods

### 2.1. Mushroom mycelium and media composition

The mushroom mycelium of Malaysian *Ganoderma lucidum* strain QRS 5120 was obtained from Functional Omics and Bioprocess Development Laboratory, Institute of Biological Sciences, Faculty of Science, Universiti Malaya as identified by Supramani et al., (2019b). The media composition was 39 g/L of potato dextrose agar (PDA, Sigma-Aldrich, Dorset, UK) powder for the plate subculture and seed culture, and the fermentation media were g/L glucose 30.0, yeast 1.0, KH_2_PO_4_ 0.5, K_2_HPO_4_ 0.5, MgSO_4_ 0.5 and NH_4_CL 4.0. All these steps were done at a temperature of 30 °C.

### 2.2. Fermentation in shake flask

The method used for inoculum preparation involving two-seed culture stages which both stages cultivated at 30 °C with an initial pH of 4 and 100 rpm for ten days and 11 days, respectively. For the first seed culture, three mycelial agar square had been cut from the PDA plate at day ten and were inoculated into 100 mL medium (30 mL glucose, 50 mL mixed media, and 20 mL distilled water) in 250 mL Erlenmeyer flask. For the second seed culture, 20% (v/v) of the mycelium from the first seed culture had been taken after being homogenized using the blender for 10 seconds to produce additional growing hyphae tips. This then being transferred into a new medium in 250 mL Erlenmeyer flask with 100 mL of the total working volume and the cultivation being carried out with pH 4, 100 rpm at 30 °C (Incubation Shaker, Multitron Pro, INFORS HT, Switzerland) before proceeding for repeated-batch fermentation.

### 2.3. Repeated-batch fermentation (RBF)

**Figure 1.**
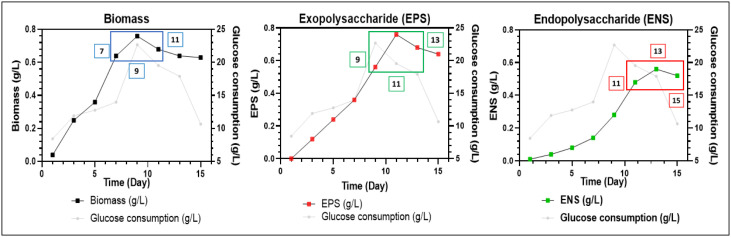
Batch fermentation growth curves and glucose consumption for the generation of broth replacement time point (BRTP) for biomass 7-9-11 (left blue box), exopolysaccharide (EPS) 9-11-13 (middle green box) and endopolysaccharide (ENS) 11-13-15 (right red box) of *Ganoderma lucidum* strain QRS 5120.

Two main factors are involved in RBF, namely, broth replacement ratio (BRR) and broth replacement time point (BRTP). The selected BRR for this study was 60%, 75% and 90%, and BRTP varied for each different response. BRTP for the biomass was day 7, 9 and 11, exopolysaccharide (EPS) were day 9, 11 and 13 and endopolysaccharide (ENS) were day 11, 13 and 15. To perform RBF, a certain amount of medium, following the BRR stated above, was taken out from each shake flask by pouring it into the measuring cylinder and replaced with an exact amount of medium at the specific BRTP. BRTP was performed by following the growth curve and production rate, as shown in Figure 1 [13]. Previous work by Supramani et al., 2019b [14] had optimized pH, glucose concentration and agitation speed for production of biomass, EPS and ENS.

### 2.4. Analytical methods

#### 2.4.1 Mycelium biomass

A sample from each shake flask was extracted by following the respective BRR. To maintain homogeneity, the medium was swirled before sampling. The sample was filtered using a Buchner funnel filter paper 0.45 µm (Whatman, Sigma–Aldrich, Dorset, UK) and the mycelial biomass was washed (3x) using sterile distilled water. Then, the mycelial biomass on the filter was subjected to food dehydrator (35 °C) until constant weight. By subtracting the weight of pre-dried filter paper from the weight of filter paper with mycelial biomass, the mycelial biomass was calculated. The concentration (g/L) was obtained by multiplying the mycelial biomass to dilution factor.

#### 2.4.2 Exopolysaccharide (EPS)

The filtered mycelial culture from Section 2.4.1 was transferred to a 15-mL centrifuge tube and the mixture was centrifuged at 10,000 rpm for 15 minutes. Resulting precipitate was discarded and the remaining supernatant was harvested for crude EPS precipitation (4). A 95% cold ethanol shock-treatment at 5 °C was added at 1:4 ratios and stored overnight at 4 °C. The precipitated EPS was re-centrifuge twice at 15,000 rpm for 20 minutes, filtered using pre-dried and weighed GF/C filter paper (Whatman Ltd., Kent, UK). EPS-filter paper combination was dried in a food dehydrator until a constant weight was observed for EPS calculation in g/L (14).

#### 2.4.3 Endopolysaccharide (ENS)

ENS from the *G. lucidum* strain QRS 5120 was extracted from the dried mycelium (biomass from Section 2.4.1). Dried mycelium (DM) was mixed with distilled water (1:10 g/mL) after the mycelial biomass concentration was calculated. Then, the distilled water containing the DM was subjected to hot water extraction method and sterilized at 121 °C for 30 minutes [15]. DM solution was filtered, and the supernatant was used for the extraction of ENS. Four volumes of 95% (v/v) ethanol was added to one volume of the supernatant from the DM solution and left overnight at 4 °C to precipitate the ENS. Subsequently, the precipitate was filtered through pre-dried and weighted filter paper and reassigned to the food dehydrator until the constant weight obtained.

#### 2.4.4 Kinetics calculation

The *G. lucidum* repeated-batch fermentation kinetic parameters were calculated as follows [16];

X max = maximum cell concentration achieved at stationary phase

X o = initial cell concentration at inoculation

$$\text{Biomass productivity}\left(\text{g}/{\text{L day}}^{-\text{1}}\right)=\frac{\text{X max}–\text{X o}}{\text{Time for product recovery }(\text{Day})}$$ (1)

$$\text{Yield}(\text{biomass})=\frac{\text{X max}–\text{X o}}{\text{Glucose consumed}}$$ (2)

$$\text{EPS productivity }\left(\text{g}/{\text{L day}}^{-\text{1}}\right)=\frac{\text{EPS max}–\text{EPS o}}{\text{Time for product recovery }(\text{Day})}$$ (3)

$$\text{Yield}(\text{EPS})=\frac{\text{EPS max}–\text{EPS o}}{\text{Glucose consumed}}$$ (4)

$$\text{ENS productivity}\left(\text{g}/{\text{L day}}^{-\text{1}}\right)=\frac{\text{ENS max}–\text{ENS o}}{\text{Time for product recovery }(\text{Day})}$$ (5)

$$\text{Yield}(\text{ENS})=\frac{\text{EPS max}–\text{EPS o}}{\text{Glucose consumed}}$$ (6)

#### 2.4.5 Residual glucose determination

The DNS method was used to estimate the residual glucose concentration based on the standard glucose calibration plot [17]. The filtered media was centrifuged at 10000 rpm for 10 minutes, and 3 mL of supernatant was transferred into a test tube using the pipette. Add 3 mL of DNS reagent to the sample and mixed properly. The test tubes were placed in a boiling water bath for 15 minutes. Then, 1 mL of Rochelle salt solution was added to stabilize the red brick colour formed. This is followed by cooling at room temperature using tap water. The absorbance of samples was measured at 575 nm using the UV-Vis spectrophotometer (Model 10S GENESYS™ UV-Vis, Thermo Scientific™).

### 2.5. Image analysis

An inverted microscope LEICA (Model DFC295, Leica Microsystems (SEA) Pte Ltd, 12 Teban Gardens Crescent, Singapore) with a coupled camera (JVC, TK-C1381 Colour Video Camera, Friedberg, Germany) was used to evaluate the morphological details of the randomly collected pellets. About 20 mL of the sample was taken from each flask and transferred into a petri dish. From each sample, three pellets were selected at random and put onto the slide to be observed under the inverted microscope for further morphological perception and analysis. The pellet diameters (horizontal and vertical) were measured using a microscopic objective stage micrometre calibration slide (10 mm/100 0.1 mm C7), at 2X magnification.

### 2.6. Characterization of β-glucan using Fourier transform infrared spectroscopy (FTIR)

FTIR analysis was performed using Agilent Cary 630 equipped with diamond ATR (attenuated total reflectance). A BG sample (0.5 g) was placed on a clean window, and the pressure clamp was closed until a click was heard. Then, the data was collected using MicroLab software (Agilent, Santra Clara, CA, USA).

### 2.7. Proton nuclear magnetic resonance (^1^H-NMR)

The NMR analysis was performed using 600 Mhz (Agilent, Santra Clara, CA, USA). BG (10 mg) was mixed with 500 µL of deuterium oxide (D_2_O). Upon dissolving, the mixture was centrifuged at 10,000 x g for 10 min. The clear supernatant was transferred to a 5 mm NMR tube (Norell, Sigma–Aldrich, Gillingham, Dorset, UK). The comparison standard used for ^1^H-NMR was laminarin (*Laminaria digitata*; Sigma–Aldrich, Gillingham, Dorset, UK). The experiment was conducted at 25 °C. A pre-saturation pulse sequence (PRESAT) experiment was performed to remove the large-signal for the HOD to determine ^1^H-NMR spectra.

### 2.8. MTT colourimetric assay

Using freshly prepared complete medium grown in a cell culture incubator (5% CO_2_, gaseous composition 95% air, 37 °C), two cell lines were used which were normal gingival cell line (hGF) and a cancerous Asian cellosaurus cell line (ORL-48). Initially, 96-well microtiter plates were seeded with hGF and ORL-48 at 2 × 104 cells/mL or each well. Both cells were permitted to cultivate 24 hours beforehand and then treated with BG at different concentrations ranging from 0 mg/mL (Control) to 4 mg/mL for the following 72 hours. The spent media was removed, and 50 µL of serum-free media and 50 µL of 3-(4,5-dimethylthiazol-2-yl)-2,5-diphenyl tetrazolium bromide (MTT) solution were added into each well and then, the plate was then incubated at 37 °C. After incubation for 3 hours, 150 µL of MTT solvent was added into each well. The plate was wrapped in foil and mixed on an orbital shaker for 15 minutes. The absorbance was read at 590 nm wavelength within 1 hour. Cytotoxicity of the MTT assay was observed as IC_50_ value half-maximal inhibitory concentration.

## 3. Results and discussions

### 3.1. Determination of broth replacement time point and broth replacement ratio

The batch fermentation growth curves for biomass, EPS and ENS production of *G. lucidum* strain QRS 5120 were performed for up to 15 days to generate each suitable broth replacement time point (BRTP), categorized as the end of log phase, transition phase and early stationary phase. In Figure 1, the BRTP was chosen at the transition phase, which was the highest peak of each bioproduct formation, according to Wan-Mohtar et al., 2016 [5] BRTP for biomass production reached the end of the log phase at day 7, followed by day 9 of the transition phase, and slowed down into early stationary phase at day 11. Meanwhile, BRTP for EPS production reached the end of the log phase at day 9, followed by day 11 of the transition phase, and slowed down into early stationary phase at day 13. This is similar with the study reported by Diamantopoulou et al., (2012) where the maximum quantity of EPS were detected in the earlier growth phases or in the early stationary phase (8th–12th day after inoculation) and this quantity seems to be reduced thereafter [18],[19]. The slight delay occurred for BRTP of ENS production as it reached the end of the log phase at day 11. Taken together, BRTP of *G. lucidum* batch fermentation showed biomass (day 7, 9 and 11), EPS (day 9, 11 and 13) and ENS (day 11, 13 and 15), respectively. According to Athenaki et al., (2018), the lower value of ENS shown at early growth phase and relatively elevated at the late growth phase which the reduction of ENS was accompanied by a gradual increase in total cellular lipids and the elevation of ENS value means that the lipid values drastically decreased at the late growth phases [20]. The same study suggested that there was relationship between the production of ENS and lipids. However, the current study only focusses on the triple production of biomass, EPS and ENS only.

For broth replacement ratio (BRR), the percentage was in the range of 75 to 90% due to removal of media less than 50%, which resulted in a higher level of waste accumulation and autolysis.

### 3.2. Pellet morphology and diameter of G. lucidum for biomass production in RBF

**Figure 2.**
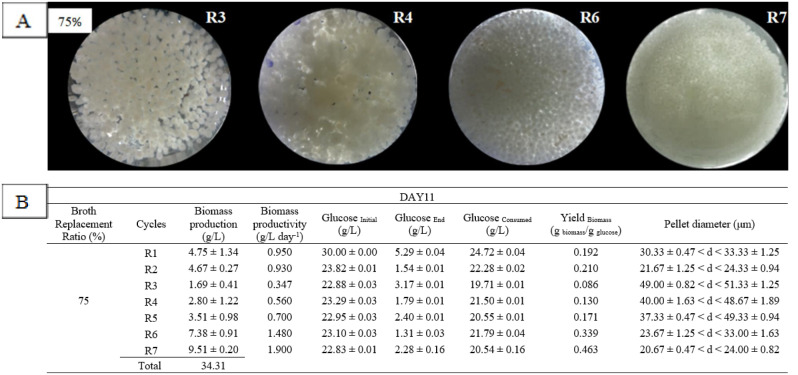
Pellet morphology (A) and diameters (B) of *G. lucidum* QRS 5120 for biomass production in repeated-batch fermentation at broth replacement time point (BRTP) Day 11 and broth replacement ratio (BRR) of 75% using initial media pH 4, 100 rpm, temperature 30 °C, and at 5-day interval between each cycle. All other fermentation media compositions were all the same [(g/L): Glucose 30, KH_2_PO_4_ 0.5, K_2_HPO_4_ 0.5, MgSO_4_7H_2_O 0.5, YE 1, NH_4_Cl 4]. R1–R7 means fermentation repetition in cycles.

The adaptation of the culture and the growth condition were observed during the RBF cycle from R1 to R7, where the RBF was performed for biomass production on day 9, and 11, as highlighted in Figure 1 (left blue box). As shown in Figure 2A, the 75% broth replacement ratio at time point day 11 started (R1) with thick-branched pellet formation. The thick branch started to detach from the mother pellet producing a second-generation pellet (hairy pellet) at R2. The surface of the hairy pellet began to increase at R4 and continue to disperse at R6. Formation of starburst-like pellet indicates a new cycle of culture, along with continuous replacement of old media with the new one at R6, and the small-compact pellets were formed at R7. Formation of hairy like structure around the pellet during the seven cycles of RBF indicated the continuous growth of fungal pellet and the new environment created by the replacement of media made the growth of the culture varied [4].

In Figure 2B, the biomass production and pellet diameter are shown. We observed that the highest biomass production (34.31 g/L) was given in BRR of 75% and BRTP at day 11 (Table 1) followed by biomass production (32.38 g/L) in BRR of 90% and BRTP at day 11. The least biomass production (12.02 g/L) was given in BRR of 60% and BRTP at day 11. From the results, we can observe RBF gave high biomass production in the early stationary phase (day 11) with small-compact pellet formation. Production of biomass decreased from R1 to R3 and started to increase from R4 till R7. We can observe the growth trending, R4 recorded the biggest pellet diameter size with the lowest biomass production, while R7 gave the highest production of biomass with small diameter pellet. The highest biomass (9.51 ± 0.2 g/L) was produced in cycle R7 with pellet diameter (49.00 ± 0.82< d <51.33 ± 1.25) µm, which was recorded to be most significant for biomass production in BRR 75%, BRTP at day 11. The compact pellet structure gave high biomass production, which was in agreement with the work done using *Ganoderma pfeifferi* by Supramani et al., 2019a [4].

### 3.3. Pellet morphology and diameter of G. lucidum for EPS production in RBF

RBF was performed for EPS production at day 9, 11 and 13, as shown in Figure 1 (green middle box). Based on the factors involved (BRTP and BRR) in repeated-batch fermentation, the highest EPS production was at BRTP day 13 and broth replacement ratio 75% with a total production of 4.34 g/L for seven cycles (R1–R7).

**Figure 3.**
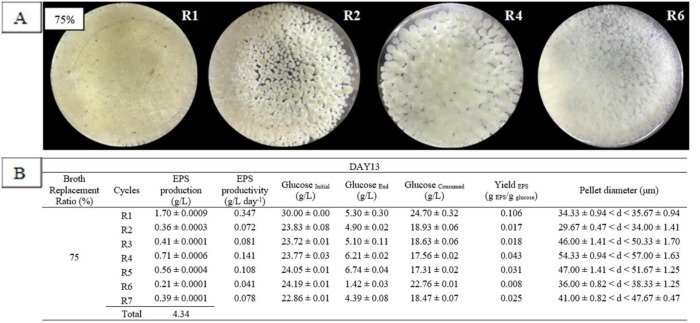
Pellet morphology (A) and diameters (B) of *G. lucidum* QRS 5120 for exopolysaccharide (EPS) production in repeated-batch fermentation at broth replacement time point (BRTP) Day 13 and broth replacement ratio (BRR) of 75% using initial media pH 4, 100 rpm, temperature 30 °C, and at 5-day interval between each cycle. All other fermentation media compositions were all the same [(g/L): Glucose 30, KH_2_PO_4_ 0.5, K_2_HPO_4_ 0.5, MgSO_4_7H_2_O 0.5, YE 1, NH_4_Cl 4]. R1–R7 means fermentation repetition in cycles.

The morphological changes of *G. lucidum* during RBF for EPS production are shown in Figure 3, indicating that pellet diameters were gradually increased from R1, R2 and R3, with the largest pellet in R4 (54.33 ± 0.94 < d <57.67 ± 1.63) µm. There was a slightly decreased pellet size at R5 and R6 before increasing again at R7. Figure 3A indicates that second-generation dispersed pellets from R1 (initial cycle) transformed to small hairy-pellets (starburst like appearance) with a few first-generation pellets (indicated by the yellow-brown colour) at R1. The formation of starburst-like pellet decreased at R2 with a few formations of a smooth-bigger pellet. The size of the pellet got bigger up to R4, with the formation of a thick-branched pellet. The thick branch from the pellet started to detach from the mother pellet to form a new pellet generation at R5 and R6. The formation of thick-branched feathers was detected at R6 and looked like a dispersed pellet up to R7. The growth for each cycle varied, as it involved the substitution of the old media with the new one, thus providing a new environment and continuous nourishment for the fungal culture.

In Figure 3B, EPS production and pellet diameter are shown for BRR of 75% and BRTP at day 13, which resulted in the highest EPS production compared to other BRTP and BRR. The production of EPS at R1 gave the highest production of EPS with 1.70 ± 0.0009 g/L and pellet diameter (34.33 ± 0.94 < d <35.67 ± 0.94) µm. At this time point and ratio, EPS favoured an ovoid-starburst pellet. Such findings were in agreement with the previous work by Wan-Mohtar et al., 2016 [4] reported that *G. lucidum* EPS production was the highest upon the formation of ovoid pellets. Another work by Supramani et al., 2019a [4] also stated that small-dispersed pellet gave the highest EPS production. Formation of starburst (hairy appearances) around the pellet promoted the production of EPS, as it was secreted in the outer layer of mycelial pellet structure [4].

### 3.4. Pellet morphology and diameter of G. lucidum for ENS production in RBF

**Figure 4.**
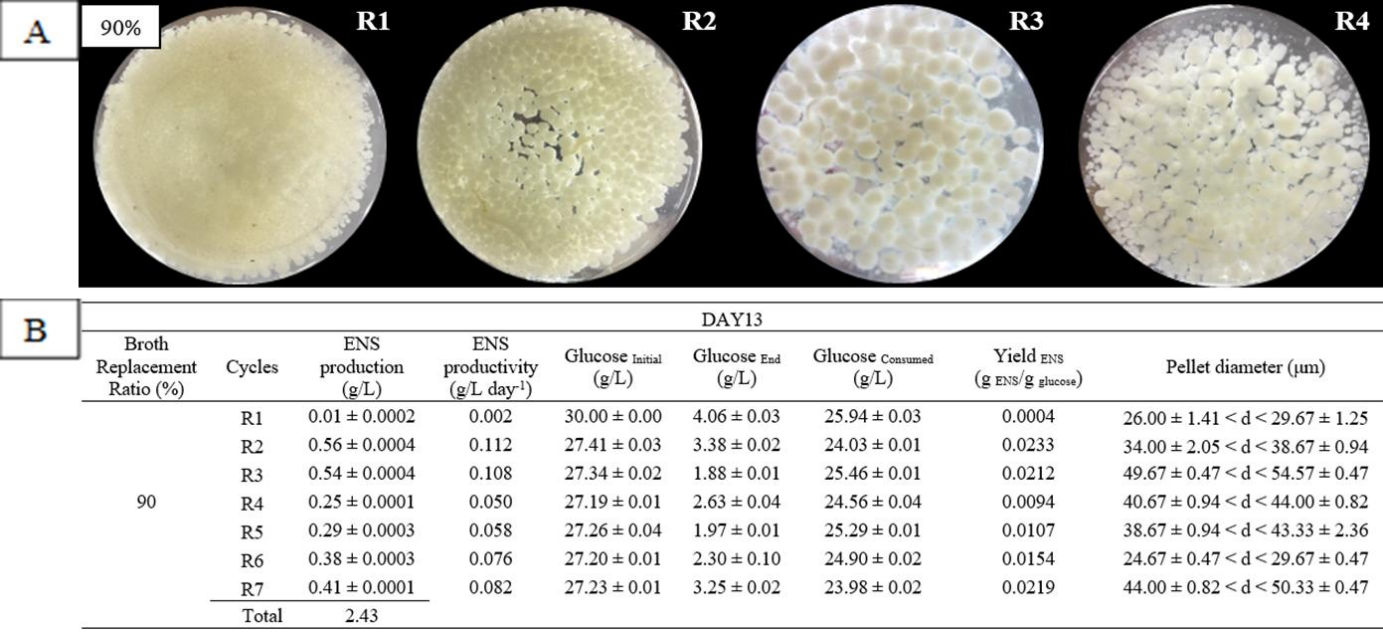
Pellet morphology (A) and diameters (B) of *G. lucidum* QRS 5120 for endopolysaccharide (ENS) production in repeated-batch fermentation at broth replacement time point (BRTP) Day 13 and broth replacement ratio (BRR) of 90% using initial media pH 4, 100 rpm, temperature 30 °C, and at 5 = day interval between each cycle. All other fermentation media compositions were all the same [(g/L): Glucose 30, KH_2_PO_4_ 0.5, K_2_HPO_4_ 0.5, MgSO_4_7H_2_O 0.5, YE 1, NH_4_Cl 4]. R1–R7 means fermentation repetition in cycles.

The highest ENS production by RBF was at BRTP day 13, which was during the transition phase and BRR 90%, with a total production of 2.43 g/L for seven cycles (R1–R7). Based on Figure 4A, the pellet had dispersed at the first cycle (R1) to generate the starburst-like pellet and become a large-smooth pellet at R2. The size of the pellet increased at R3 with the formation of dense pellet, and the culture became cloudier (under macroscopic observation) than the previous cycle. The thick-branched pellet with a few clumped feathers formed at R4 indicated that two different generations of pellet formed inside the flask and the branch started to detach and became denser.

Figure 4B shows the diameter of pellet for production of ENS at BRR of 90% and BRTP at day 13 that produced the highest yield of ENS using RBF strategy. In the second cycle, the highest ENS production, which was 0.56 ± 0.0004 g/L, was produced with a diameter range (34.00 ± 2.05< d < 38.67 ± 0.94) µm. At this time point and ratio, ENS favours large-hollow pellet as the ENS was produced inside the mycelium [14].

### 3.5. Pellet morphological comparison of biomass-EPS-ENS production of G. lucidum in RBF

**Figure 5.**
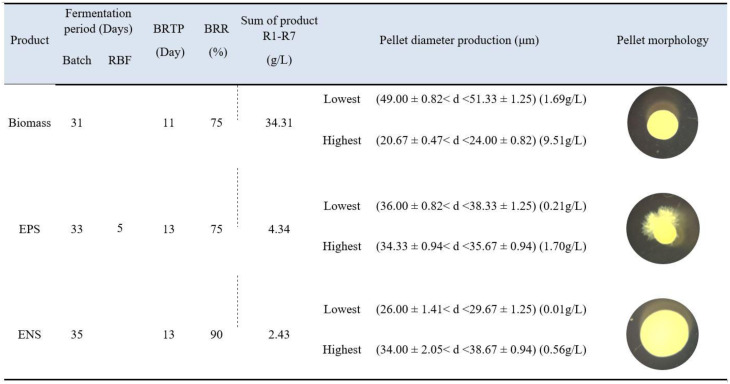
Comparison for production and pellet morphology of biomass, exopolysaccharide (EPS) and endopolysaccharide (ENS) for *G. lucidum* QRS 5120 in repeated-batch fermentation. *BRTP = broth replacement time point, BRR = broth replacement ratio, R1 – R7 = fermentation repetition in cycles.

As shown in Figure 5, each biomass, EPS and ENS productivity favours different pellet morphological characteristics. For high biomass production, it favoured a small-compact pellet with a mean diameter of 22.34 µm, while ovoid-starburst pellets with a mean diameter of 35.0 µm favoured high EPS production. Meanwhile, large-hollow pellets with a mean diameter of 36.34 µm favoured high ENS.

Figure 5 also represents the comparison of each biomass, EPS and ENS production in RBF for *G. lucidum* QRS 5120 mycelium in the shake flask. For the fermentation period, the batch took 31, 33 and 35 days for one run, starting from the initial subculture phase. Nonetheless for RBF, it provided continuous production in an interval of five days only (up to seven cycles), which shortened the time. Moreover, Wan-Mohtar et al., 2016 [5] did six-day intervals for each RBF cycle for *G. lucidum* BCM 31549. Remarkably, this strain successfully did a 5-day interval, thus making the fermentation time shorter. The strain studied by Wan Mohtar et al., 2016 [13] survived until the fifth cycle, and after the fifth cycle, the autolysis event took place, indicated by the colouration of pellet to yellowish-brown. For this strain, it managed to survive until the seventh cycle, which was more productive and showed that *G. lucidum* cells demonstrated robustness to the RBF process [5]. The cycles had to stop at R7 due to pellet colour changes (indicating autolysis behaviour) and potential toxic metabolite build-up that may have disrupted the morphology [5].

### 3.6. Pellet morphological comparison of the current work on G. lucidum for biomass-EPS-ENS production in RBF based on available literature

Table 1 shows the available reported studies on the pellet diameter and morphological characteristics for the *Ganoderma* species using a batch fermentation technique for biomass-EPS-ENS production. The comparison shows that all the studies did not report on the high potential of ENS production [15] compared to the current work. All reported studies demonstrated the relationship between pellet morphology and high EPS production in a fermentation process, whereas the current study highlighted the triple production of biomass, EPS and ENS for Malaysian *Ganoderma lucidum*. Supramani et al., 2019b [4] and Wan-Mohtar et al., 2016 [5] indicated that both *G. pfeifferi* and *G. lucidum* BCM 31549 showed resilience to shear stress and marked adaptability to the RBF strategy, with high survivability for sustainable EPS production in an extended fermentation strategy. The current study presents the pellet morphology and diameter for production of not only the EPS but also biomass and ENS (large-hollow pellet) in repeated-batch fermentation. To our knowledge, there is no work has been done for the production of ENS using RBF technique. Moreover, the production of EPS using RBF was the highest compared to other studies.

**Table 1.** Comparison of the current work on *Ganoderma sp*. fermentation regarding biomass, exopolysaccharide (EPS) and endopolysaccharide (ENS) production and its morphology.

*Ganoderma sp.*	Mode	Flask/ bioreactor	Initial glucose concentration (g/L)	Agitation speed (rpm)	Product	Production (g/L)	Pellet morphology	Pellet diameter (µm)	References
*G. lucidum* QRS 5120	RBF	250 mL flask	30	100	Biomass	34.31	Small-compact pellet	20.67 < d < 24.00	*Current work*
					EPS	4.34	Ovoid-starburst pellet	34.33 < d < 35.67	
					ENS	2.43	Large-hollow pellet	34.00 < d < 38.67	
*G. pfeifferi* IMI 379841	Batch	250 mL flask	9	120	Biomass	3.63	Large-compact pellet	40.00 < d < 40.67	[4]
					EPS	0.65	Small disperse pellet	14.33 < d < 16.00	
					ENS	NA	NA	NA	
*G. lucidum* BCM 31549	RBF	500 mL flask	50	100	Biomass	1.62	NA	NA	[5]
					EPS	0.20	Ovoid pellet		
					ENS	NA	NA		
*G. lucidum* CCRC36123	Fed-batch	15 L bioreactor	35	300	Biomass	26.60	NA	NA	[21]
					EPS	4.55	Freely dispersed and clumped pellets		
					ENS	NA	NA		
*G. lucidum*	Batch	500 mL flask	40	150	Biomass	5.41	NA	NA	[22]
					EPS	2.63	Slight roughness around the edge pellets	800 ≤d < 2500	
					ENS	NA	NA	NA	
*G. applanatum* AMRL 341	Batch	100 mL flask	30	120	Biomass	13.34	Looser masses of hyphae	NA	[19]
					EPS	0.55			
					ENS	5.89			
*G. lucidum* strain Ga. l 4	Batch	10 L bioreactor	20	100	Biomass	10.00	Spherical pellets	NA	[23]
					EPS	0.68			
					ENS	3.30			
*G. lucidum* CCGMC 5.616	Batch	250 mL flask	35	120	Biomass	15.50	NA	< 10000	[24]
					EPS	0.63			
					ENS	1.06			

### 3.7. FTIR analysis of EPS and ENS

**Figure 6.**
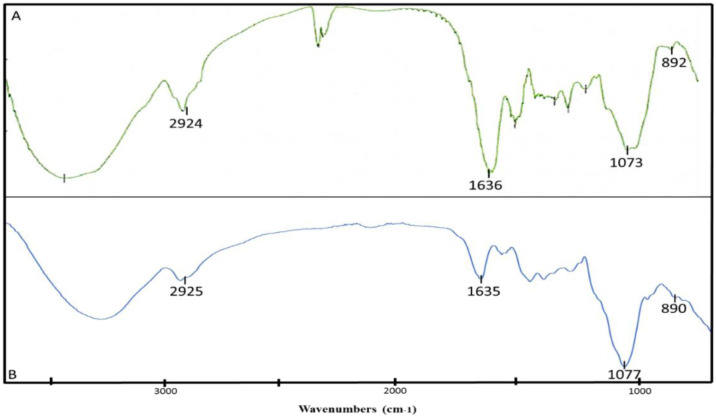
FTIR spectra result of β-glucan (A: crude exopolysaccharide (EPS), B: crude endopolysaccharide (ENS)) from the mycelium *G. lucidum* QRS 5120 in a repeated-batch fermentation.

FTIR spectroscopy was used to identify the position and anomeric configuration of the glycosidic linkage in the glucan of the targeted EPS-ENS extracts from *G. lucidum* in Figure 6 (A = EPS, B = ENS[25]. Overall, the absorption in the region between 1250 cm^−1^ and 1650 cm^−1^ indicated the sample was a polysaccharide [26]. The strong, broad peak in the absorption region between 3000 cm^−1^ and 3500 cm^−1^ indicated the stretching vibration of O–H groups in the sugar residue as well as showing the presence of polyhydroxilic compounds [10],[27]. The absorption peaks at 2924 cm^−1^ in A and 2925 cm^−1^ in B were associated with the stretching vibration of C–H in the sugar ring, indicating the presence of a methylene group, –CH_2_ [2]. The absorption peak in A (1636 cm^−1^) and B (1635 cm^−1^) showed the water bending vibration in the polysaccharide [2],[28]. The absorbance at 1073 cm^−1^ in A and 1077 cm^−1^ in B also shows the presence of C–O–C and C–O bonds stretching vibrations. Furthermore, the presence of C–O, C–O–C and O–H stretching vibrations absorption peaks resembles the characteristic of a polysaccharide structure [2],[10]. Besides, according to Usuldin et al., 2020 [2], the finger-print region from 850 cm^−1^ to 1000 cm^−1^ is able to determine the type and anomeric configuration of the polysaccharide, and Wan-Mohtar et al., 2016 [10] also reported that the absorption region between 700 cm^−1^ to 950cm^−1^ is for the anomeric region. The absorbance at 890 cm^−1^ in A and 892 cm^−1^ in B confirmed that the anomeric configuration of the glycosidic linkage was in β configuration [2],[29]. Thus, based on the absorption peaks, it could be summarised that the structural characteristics of the polysaccharide (both from EPS and ENS) from the mycelium *G. lucidum* was a β-glucan.

### 3.8. ^1^H-NMR spectroscopic analysis of EPS and ENS

**Figure 7.**
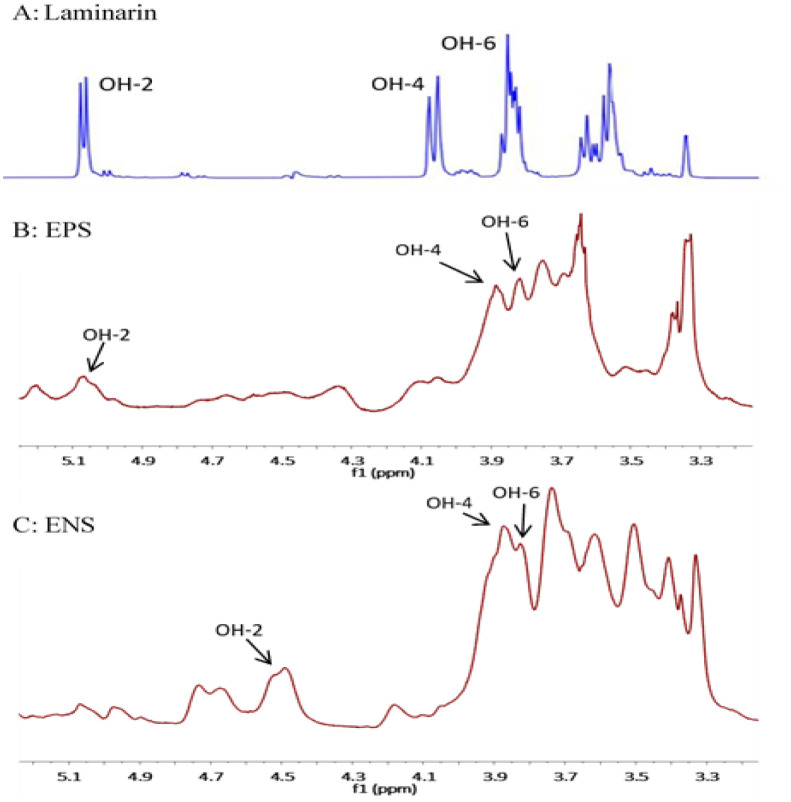
^1^H-NMR spectra of β-D-glucan (BG) of (A) laminarin (*Laminaria digitata*) standard, (B) exopolysaccharide (EPS) and (C) endopolysaccharide (ENS) derived from *G. lucidum* QRS5120 mycelium in a repeated-batch fermentation.

As can be seen in Figure 7, ^1^H-NMR spectroscopic analysis of the β-glucans of EPS and ENS from *G. lucidum* was conducted to verify the presence and the structure of mycelial β-glucan at 24 °C using D_2_O as a solvent. The result was compared to laminarin, a standard for (1→3)-β-D-glucan. All of the resonated signals obtained for both glucans were in the downfield region and also produced a signal in the range of 4.3 ppm to 5.6 ppm, which is the signal for the anomeric proton of β-configuration [25],[30]. The resonance signals with the chemical shift of 3.87 ppm and 3.81 ppm were assigned for proton OH-4 and OH-6, respectively, which was similar to the standard laminarin, while the signals of EPS at 5.1 ppm and ENS at 4.5 ppm were assigned to OH-2, the anomeric proton [2],[4]. This was agreed by Wan-Mohtar et al., 2016 [10] where they reported that the glucans of *G. lucidum* had the spectrum chemical shifts between 3.9 ppm and 5.4 ppm. Therefore, based on the FTIR (Figure 6) and ^1^H-NMR results (Figure 7) proved that the glucans EPS and ENS were likely composed of (1-3)-β-D-linkages (a typical structure of β-glucan), as mentioned by Supramani et al., 2019b [4].

### 3.9. Anti-oral cancer

**Figure 8.**
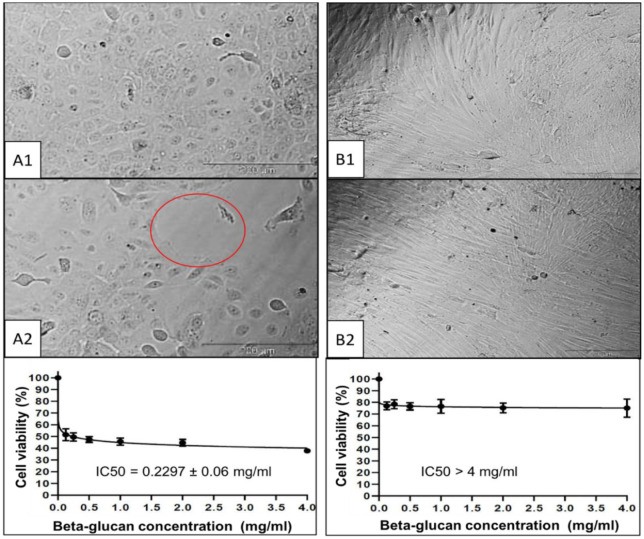
Cytotoxic effect and IC_50_ value (half maximal inhibitory concentration) of beta-glucan (BG) from liquid fermentation of *Ganoderma lucidum* QRS 5120 mycelium against oral cancer cell line (ORL-48) (Figure A1; 0 mg/mL and A2; 4 mg/mL) and normal gingival cell line (hGF) (Figure B1; 0 mg/mL and B2; 4 mg/mL). Red circle (Figure A2) shows the free matrix in the media due to a decrease in cells. Bar = 20 µm.

To evaluate the bioactive potential of BG from one of the extracts, only the cytotoxic effect of EPS-BG from *G. lucidum* was tested against the normal gingival fibroblast cell line (hGF) and oral cancer cell line (ORL-48). Figure 8 shows the morphological observation of hGF cells (Figure 8B1 and 8B2) and ORL-48 cells (Figure 8A1 and 8A2) upon 72 hours of treatment with EPS-BG at 0 mg/mL and 4 mg/mL. The EPS-BG showed antiproliferative activity against ORL-48 cells, as Figure 8A2 showed a reduction in cell growth compared to control (Figure 8A1). In contrast, no change was observed in the cell viability for the hGF cell line. The half-maximal inhibitory concentration (IC_50_) for hGF cell line were IC_50_ > 4 mg/mL, which suggest that BG is safe for normal cells, but ORL-48 cell line showed an IC_50_ of 0.23 mg/mL, which indicates that BG is more cytotoxic towards cancer cells. It can reduce their proliferation, as suggested by Zeng et al., 2020 [31]. A lower IC_50_ value demonstrated higher toxicity and vice versa [32]. The tested EPS-BG concentration (<4 mg/mL) was considered low, yet effective against both normal and cancerous cell line as in agreement with the same EPS-BG from French-originated *G. lucidum* (60 µg/mL) [10]. Thus, such BG could be suggested as a high potential natural drug compound.

## 4. Conclusions

For each biomass, EPS and ENS production favoured a different pellet diameter and morphological characteristics for *G. lucidum* when grown in a repeated-batch fermentation. Biomass (34.31 g/L) production favoured small-compact pellets with a diameter range of 20.67 µm < d < 24.00 µm. Meanwhile, EPS (4.34 g/L) production favoured ovoid-starburst pellets with a diameter range of 34.33 µm < d <35.67 µm and ENS (2.43 g/L) favours large-hollow pellets with a diameter range of 34.00 µm < d <38.67 µm. *G. lucidum* pellets successfully sustain extended fermentation in RBF up to seven cycles. The structure of a crude polysaccharide from the mycelium of *G. lucidum* was β-glucan in the form of (1-3)-β-D-linkages for EPS and ENS. The extracted EPS β-glucan possesses anti-oral cancer activity.
